# Predicting large wildfires across western North America by modeling seasonal variation in soil water balance

**DOI:** 10.1007/s10584-015-1569-x

**Published:** 2015-12-03

**Authors:** Richard H. Waring, Nicholas C. Coops

**Affiliations:** College of Forestry, Oregon State University, Corvallis, OR USA; Department of Forest Resource Management, University of British Columbia, 2424 Main Mall, Vancouver, Canada

## Abstract

A lengthening of the fire season, coupled with higher temperatures, increases the probability of fires throughout much of western North America. Although regional variation in the frequency of fires is well established, attempts to predict the occurrence of fire at a spatial resolution <10 km^2^ have generally been unsuccessful. We hypothesized that predictions of fires might be improved if depletion of soil water reserves were coupled more directly to maximum leaf area index (LAI_max_) and stomatal behavior. In an earlier publication, we used LAI_max_ and a process-based forest growth model to derive and map the maximum available soil water storage capacity (ASW_max_) of forested lands in western North America at l km resolution. To map large fires, we used data products acquired from NASA’s Moderate Resolution Imaging Spectroradiometers (MODIS) over the period 2000–2009. To establish general relationships that incorporate the major biophysical processes that control evaporation and transpiration as well as the flammability of live and dead trees, we constructed a decision tree model (DT). We analyzed seasonal variation in the relative availability of soil water (*fASW*) for the years 2001, 2004, and 2007, representing respectively, low, moderate, and high rankings of areas burned. For these selected years, the DT predicted where forest fires >1 km occurred and did not occur at ~100,000 randomly located pixels with an average accuracy of 69 %. Extended over the decade, the area predicted burnt varied by as much as 50 %. The DT identified four seasonal combinations, most of which included exhaustion of ASW during the summer as critical; two combinations involving antecedent conditions the previous spring or fall accounted for 86 % of the predicted fires. The approach introduced in this paper can help identify forested areas where management efforts to reduce fire hazards might prove most beneficial.

## Introduction

Throughout the western United States and western Canada the area burnt by wildfires has varied annually over the last three decades by nearly 10-fold (Skinner et al. 1999; Littell et al. 2009). Although regions with Mediterranean climates, characterized by dry summers and warm winters, are more prone to wildfires than those receiving summer precipitation, the patterns are not easily predicted across 34 ecoregions that contain forests (Waring et al. 2011). There seems little disagreement, however, that since the 1980s, climatic warming has extended the areas prone to fires (Westerling et al. 2006) as mountain snow packs melt earlier and the growing season is extended to higher elevation forests (Hamlet et al. 2006).

Although fire histories differ significantly among regions in western North America, recent trends in area burned are generally upward, sometimes approaching or exceeding estimates of areas burnt since Europeans settled the continent (Swetnam and Betancourt 1998; Schoennagel et al. 2004; Balshi et al. 2007; Weisberg and Swanson 2003; Fry and Stephens 2006; Riley et al. 2013; Dennison et al. 2014). Higuera et al. (2015) showed “that models developed using fire-climate relationships from recent decades over predict areas burned when applied to earlier periods.” They suggests that accumulation of fuels following a long periods of unusually low fire activity, combined with more effort to control wildfires, may have contributed to altered fire-climate relationships, a view supported by others (e.g., Marlon et al. 2012, North et al. (2015).

Littell et al. (2009) analyzed climatic controls on the area burnt since 1916, showing that different ecoregions in the western U.S. varied in their sensitivity to antecedent conditions. Westerling et al. (2002) developed a statistical method, using the Palmer Drought Severity Index (PDSI) to forecast the area burned in different forest provinces. They inferred a strong negative association with the availability of soil moisture immediately prior to the fire season in most heavily forested federal lands, while in more arid regions, the relationship was reversed, and applied up to a year earlier. A strong positive correlation between antecedent soil moisture and wildfires should be expected where the forest canopy is sparse and moist soils allow for the development of a lush understory. Such an understory, if composed of shallow-rooted grasses, forbs and shrubs, quickly dries during a dry summer and become flash fuel (Dimitrakopoulos and Bemmerzouk 2003). These high correlations between extreme weather conditions conducive to wildfires and area burned suggest that current management practices, when limited to small,isolated areas, will have little effect on the extent of wildfires as climatic conditions continue to warm (Hessburg et al. 2015). On the other hand, knowing more accurately where large fires are most likely to occur should provide policy makers and managers a rational for action to reduce the likelihood of ignition (Syphard and Keeley 2015) and damage to natural and human resources (San-Miguel-Ayanz et al. 2013).

Clearly, not all forests are equally prone to wildfire. The marine West Coast forest zone of Oregon, Washington, and northern California, which support the highest leaf area and produce the greatest accumulation of live and dead biomass (Sun et al. 2004). rarely burns (Long et al. 1998; Riley et al. 2013). The reason for the infrequency of fire in this zone is because trees normally have access to sufficient water through fog-drip, dew and moist soils so as not to become flammable (Hessburg et al. 2015) except when essentially all available water is depleted from the rooting zone (Breda et al. 2006). There is some difficulty referencing flammability to foliar moisture content (% dry mass) because non-structural carbohydrates and fats accumulate in foliage during the growing season (Jolly et al. 2012). Severe drought, associated with depletion of all available water in the rooting zone, combined with high evaporative demand, can cause foliage moisture contents to drop below 100 %-120 %, which is considered an approximate threshold below which a crown fire can be sustained (Agee et al. 2002). With further drying, the water conducting pathway of stems, roots, and branches may cavitate (Breda et al. 2006). When this happens, all tree components, as well as understory vegetation, litter and coarse woody debris, become highly flamable (Chuvieco et al. 2004).

The extent that a regional-scale extreme drought affects trees depends on their rooting depth and their density of leaves. Peterman et al. (2013) demonstrated drought-associated outbreaks of bark beetle on pinyon pine were largely concentrated on shallow soils. Unfortunately, most large-scale soil maps are inprecise (Peterman et al. 2014). Coops et al. (2012) attempted to remedy this situation by inverting a process-based growth model, constrained by satellite-derived estimates of maximum leaf area index (LAI_max_), to derive estimates of the available soil water storage capacity (ASW_max_) as well as soil fertility (S_f_) at a spatial resolution of 1 km across most of western North America. The estimates of ASW_max_ are more accurate than those of S_f_ because drought-adapted forests generally have LAI_max_ values <3.0, whereas more fertile, less drought-prone forests often have LAI_max_ > 6.0.

To estimate the state of plant dessication accurately requires refinements that are rarely included in even sophisticated hydrologic models (e.g., Elsner et al. 2010). For example, transpiration needs to be recognized as a non-linear function of LAI_max,_ which can vary interannually by >50 % (Breda et al. 2006). Below an LAI_max_ of ~5.0 m^2^ m^−2^ maximum transpiration rates are reduced linearly; whereas above that threshold, the rates plateau as progressively more leaf- shading restricts stomatal opening (Granier et al. 2000). Similarly, the depletion of available water in the rooting zone exerts non-linear constraints on stomatal conductance, and thus alters the time required to dissicate living vegetation (Sucoff 1972; Sun et al. 1995).

We hypothesize that models that include LAI_max_ and assess the implications of seasonal variation in soil water balances should improve predictions of fire occurrences over those that lack these features. In this paper, we evaluate the extent that large forest fires since the turn of the 21st Century can be predicted at a spatial resolution of 1 km based on simulated patterns of available soil moisture affected by changing climatic conditions. To accomplish this, we use the same process-based growth model, climatic data, and derived estimates of ASW_max_ previously employed by Coops et al. (2012) for the period 2000–2009. MODIS Active fire “hotspot” (MCD14DL) data with a spatial resolution of 1 km served as our reference to the location and size of wildfires; this dataset compares well with 30 m Landsat fire mapping (Hantson et al. 2013) with methods available to improve resolution at daily time steps (Parks 2014).

## Methods

In this section, we define the baseline against which modeled predictions of fire were compared, introduce the model used to make predictions of monthly depletions in available soil water and constraints on transpiration, and describe the construction of maps and statistical comparisons made to evaluate the relations between predicted and observed wildfires. Our analysis encompasses forested sites in 34 ecoregions distributed from 32.5^o^S to 60^o^N Latitude and from 110^o^W to 126^o^W Longitude. It excludes most of the Yukon Territory where peat and permafrost are more prevalent than in the rest of the study area, and thus corresponds with our previous analyses of species distribution and forest disturbance in response to recent climatic variation (Coops et al. 2011).

### Selection and processing of MODIS hotspot data

We acquired MODIS-derived (MCD14DL) active fire “hotspot” 1 km maps from 2000 through 2009 from the United States Forest Service (USFS) at 1 km resolution from across western North America (http://activefiremaps. fs.fed.us/gisdata.php). The fire-detection algorithm relies on temperatures detected at two thermal infrared wavelength that can discriminate active fires as small as 100 m^2^ at ~1000°K when viewed from nadir, and has a 50 % chance of identifying large (1–2 km^2^) smoldering fires at ~600°K (Giglio et al. 2003). MODIS imagery, which includes other spectral bands, is used to screen for false thermal signals and to mask for clouds at a spatial resolution of 250 m (Justice et al. 2002).

We selected fire event recorded for three years (2001, 2004, 2007) because these years represent, respectively, low, moderate, and high rankings in area burned during the decade 2000–2009. To minimize registration errors, all isolated (hotspot) fires of <100 ha within a 3 × 3 km area were excluded from the analysis. We further defined a set of random points within the study area for analysis that included only those masked as forests and not registered as burnt in the three selected reference years. In total, over the 3 years, 50,080 pixels were detected as active fires within forested pixels throughout the region. A commensurate number of randomly selected fire-free pixels were also selected over the three fire seasons.

### Climatic data

Mean monthly climate spatial surfaces were generated using ClimateWNA (Wang et al. 2012). The program extracts and downscales PRISM (Daly et al. 2008) and ANUSPLIN (Hutchinson 2004 generated monthly data (2.5 × 2.5 arcmin) to 1 km and calculates seasonal and annual climate variables for specific locations based on latitude, longitude and elevation. Elevation adjustments are achieved through a dynamic local regression function developed individually for each monthly climate variable in the baseline dataset. To provide the required elevation data for ClimateWNA at 1 km, a 90 m Digital Elevation Model (DEM) was resampled from the Shuttle Radar Topography Mission (SRTM). Spatial layers and point -based climate estimation are available online at: http://cfcg.forestry. ubc.ca/projects/climate-data/climatebcwna/. Mean monthly daytime vapor pressure deficits (VPDs) were estimated by assuming that the water vapor concentrations present on the day would be equivalent to those held at the mean minimum temperature (Kimball et al. 1997). The maximum mean VPD was calculated each month as the difference between the saturated vapor pressure at the mean maximum and minimum temperatures. Mean daytime VPD was calculated at two thirds of the maximum value. The number of days per month with subfreezing temperatures (≤2 °C) was estimated from empirical equations with mean minimum temperature. Subfreezing temperatures are important because they cause stomata to close, and to remain close for at least a day (Hadley 2000).

Monthly estimates of total incoming short-wave radiation were obtained by combining the synoptic and zonal variation captured by the North American Regional Re-Analysis (NARR). NARR is an improved version of the National Center for Environmental Prediction/National Center for Atmospheric Research (NCEP NCAR) Global reanalysis data may be downloaded from the www.cdc.noaa.gov. Briefly, the surface radiation balance (e.g., incoming and outgoing shortwave and longwave) is estimated using a precipitation assimilation procedure (Zhao et al. 1997) that adjusts ambient conditions to closely match precipitation measurements from a variety of sources. Down-scaling was accomplished making topographic adjustments based on an approached developed by Fu and Rich (2002) to produce 1 km radiation surfaces from the broader scale NARR layers.

### Process-based model to predict transpiration, evaporation, and soil water depletion

There are a wide variety of physiologically-based process models, but only a few have been designed to scale projections of photosynthesis, structural growth and mortality across landscapes (see the review by Mäkelä et al*.*^2000^). Among the most widely used is the 3-PG model (Landsberg and Waring 1997). The 3-PG model differs from others primarily in a number of simplifying assumptions: (1) that monthly mean climatic data are adequate to capture major trends in drought; (2) that autotrophic respiration (R_a_) and net primary production (NPP) are approximately equal fractions of gross photosynthesis (GPP); and (3) that the proportion of NPP allocated to roots increases from 25 % to 60 % as nutrients (particularly nitrogen) become progressively less available.

The model (edition 3PGpjs2.7) calculates gross photosynthesis, canopy evaporation and transpiration, growth allocation and litter production at monthly intervals (*∆t*). It reduces potential photosynthesis and transpiration by imposing restrictions on stomatal conductance through modifiers, taking values between 0 = complete restriction and 1.0 = no restriction, that account for the effects of frost, high vapor pressure deficits and limitations in available soil water content. The soil water modifier (*fASW*) is determined as a non-linear function from the ratio of the amount of water available in the root zone of the trees (*ASW*) to the maximum value (*ASW*_max_). *ASW*_max_ is the available water holding capacity of the soil, which is the difference between the water content in the root zone at field capacity and at the wilting point. For any given month, ASW is calculated from:

1 $$ ASW\left(t+\varDelta t\right)=ASW(t)+\left(P-E-T\right) $$

where *ASW*(*t*) is the value at the beginning of the month and *P*, *E* and *T* denote the monthly values of precipitation, evaporation and transpiration, respectively. The model includes a term to account for rainfall interception by the forest canopy; this water is assumed to be lost by evaporation, giving *E*. Transpiration is calculated from the Penman–Monteith equation, which incorporates a canopy conductance term derived from stomatal conductance and LAI. If the value of *ASW* on the left-hand-side of Equation (1) exceeds *ASW*_max_, i.e*.*, the whole root zone is at field capacity, the excess is assumed to be lost as runoff or drainage.

At monthly time steps, the model is unable to compute a snow water balance accurately, although one may assume that precipitation in months with average temperatures well below freezing is largely in the form of snow, but changes in albedo, incident radiation and other factors determine the accumulation and melting dates of snow (Coughlan and Running 1997; Elsner et al. 2010). At annual time steps, the model sums monthly changes in tree number, mean diameter, stand basal area, above-ground volume and biomass and updates the changes in LAI.

To account for seasonal adjustments in temperature optima (Hember et al. 2010) and to incorporate the large genetic variation among populations of Douglas-fir, we broadened the range for which photosynthesis could remain above 50 % of maximum to lie between 0 °C and 35 °C by setting minimum, optimum and maximum temperatures at −7 °C, 18 °C and 40 °C, respectively. The photosynthetic response at temperatures below −2 °C is truncated to zero because stomata are closed below this temperature threshold (Hadley 2000).

The fertility-dependent growth modifier in the 3-PG model is a function of the soil fertility rating, *S*_*f*,_ which ranges between zero, for the poorest soils, to unity for most fertile soils (Landsberg and Waring 1997). As previously mentioned, we generated estimates of S_f_ and *ASW*_max_ at 1-km resolution for all forested sites by inverting the 3-PG model to achieve close agreement between modeled LAI_max_ and MODIS-derived observations over the range from 0.5 to 6.0 m^2^m^−2^ (Coops et al. 2012). The model was parameterized for Douglas-fir with the same values used to derive estimates of soil properties (Waring and McDowell 2002; Coops et al. 2012).

### Comparison of model predictions with MODIS hotspot data

The 3-PG model predictions of *fASW* were generated for each month for the years 2000 through 2009. The monthly values were averaged for the four seasons (winter: December-February; spring: March-May; summer: June-August, autumn: September-November). We chose to apply a decision tree analysis to develop relationships between the seasonal *fASW* and the presence and absence of fire in 2001, 2004, and 2007. Decision tree models are increasingly selected for model development in ecological research because of their ability to deal with collinear datasets, to exclude insignificant variables, and to allow for asymmetrical distribution of samples (De’ath 2002, Schwalm et al. 2006; Melendez et al. 2006). They, like non-linear panel modelling and other recent statistical innovations, allow for links to be developed between environmental data and fire occurrence even with incomplete information and variable correlations (An et al. 2015).

The Decision Tree approach separates the dependent variables (seasonal *fASW*) into a series of binary (yes/no) choices that identifies, by the topography in the decision tree, if pixels have been detected as a hot spot in the MCD14DL layer. Decision Tree Regression (DTREG) software (Sherrod 2010) was used to create a decision tree using 10-fold cross validation technique where the data are separated randomly into 10 equally sized subsets and models developed on nine of the groups, and then tested against the remaining 10 %. This process of k-fold partitioning is then repeated with results merged to produce a final classification tree (Breiman et al. 1984). To assess accuracy once the model was created, a ‘confusion matrix’ was developed, which provides an indication of the positive and negative predictive power of the model as well as a number of other statistics (Fielding and Bell 1997, but see caveats in Lobo et al. 2008). Our analysis included an evaluation of the amount of variance accounted for in the model by each of the seasonal indices of *f*(*ASW*). Once developed, the model was run with the derived seasonal indices of soil water depletion to predict annual fire occurrence layers for each year over the decade (starting with data from 1999). A total of 99,930 pixels were evaluated, half of which had recorded fires and half without.

## Results

**Fig. 1:**
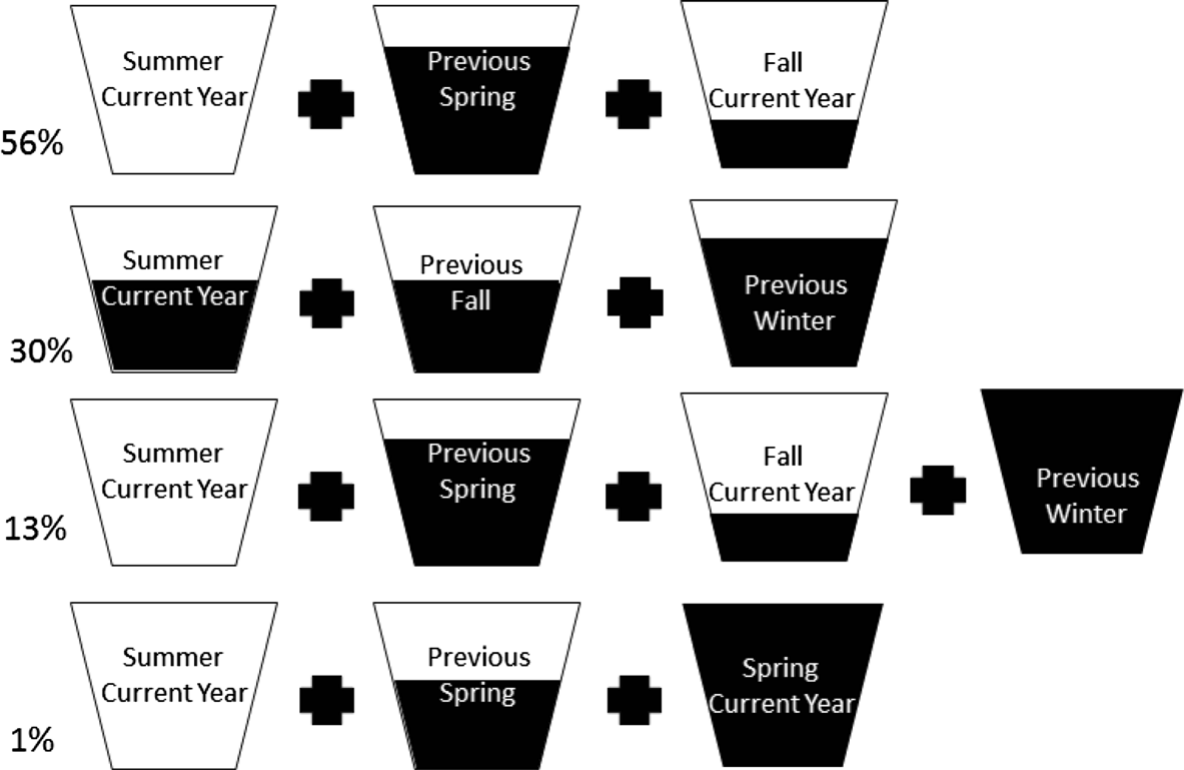
Schematic diagram showing four separate decision tree rules that specify different seasonal combinations of the function available soil water content (*f*ASW) where clear =completely depleted, all black = full capacity, others = black proportional to remaining capacity. The four rules were derived with seasonally averaged climatic data acquired across the study area in 2001, 2004 and 2007 and each of the respective previous years. The rules predict the occurrence of at least two, 1 km size fires within 3 × 3 km sampled areas. The percentages that each of the 4 rules was utilized in predicting the presence or absence of wildfires across the study area are listed on the left side of the figure

The decision tree model created four rules based on current and previous year’s seasonal patterns in *fASW* to predict the location of active fire hotspots in the three years analyzed. Three of the rules, depicted in Fig. 1, accounted for 99 % of the predicted fires. All recognize the importance of summer drought in the year of the fire as of paramount importance. The model defined a summer threshold of *f*(*ASW*) at 0.12 of optimum, below which fires would have high probability.

In the first decision rule, the indicator of summer drought is coupled with moderate drought conditions in the spring of the previous year, and drought the current fall. The second rule, unlike the 1st, predicts that sites with moderate rather than severe summer drought in the current year would still be fire prone if fall and winter conditions the year before were droughty. The third rule mirrors the first with summer drought in the current year and drought conditions in the previous fall, but recognizes that a favorable water balance the previous winter could stimulate the production of flash fuel. The last rule describes a similar pattern to the first and third with drought in the current summer, but with favorable spring growing conditions the previous year more important. Overall, the decision tree model (applying all 4 rules) accounted for 69 % of the variance in fires recorded during the three selected calibration years, (70 % with fire absent, 68 % when present).

The proportion of sites classified by each of the 4 rules varied from 1 % to 56 % with rules 1 and 2 contributing a total of 86 % (Fig. 1). We note that unlike a linear regression model, a variable in a decision tree model can be important even if it never appears as a primary splitter at a node. The importance of a variable is assessed whether it serves in a primary and surrogate role based on the amount it improves, relative to the best identified variable, the overall model predictions.

**Fig. 2:**
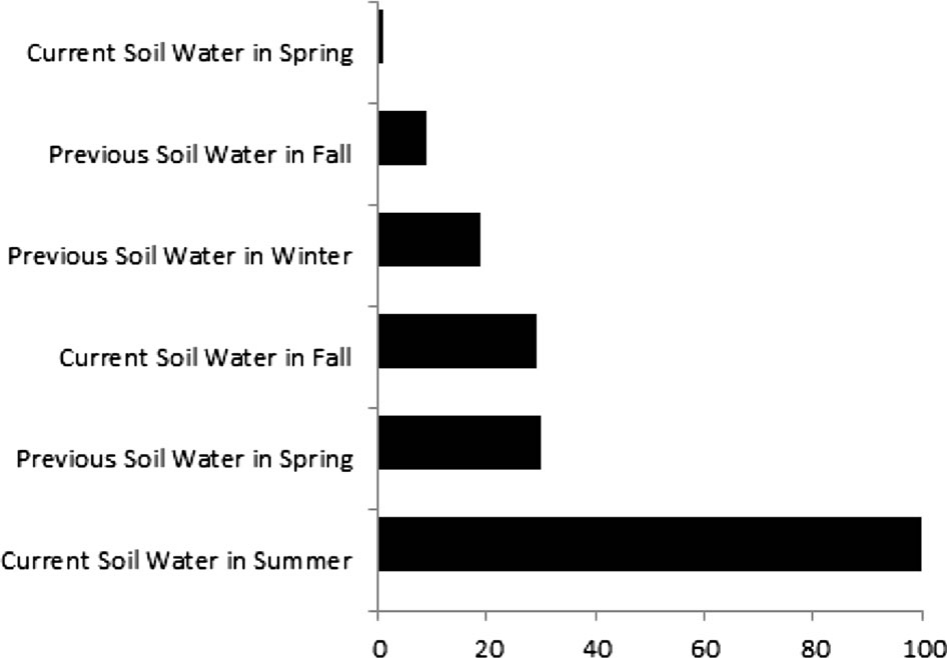
The relative importance (x-axis) of different seasonal functions of available soil water (*fASW*) (y-axis) varied in their contributions to the four rules used to predict MODIS Active hotspot occurrence of wildfires in 2001, 2004, and 2007

Although a number of seasonal *fASW* of the current and previous year were used in the decision tree analysis, their relative importance differed; Fig. 2 provides a summary of how often seasonal *fASW* modifiers were utilized in our decision-tree model. The results confirm that the *fASW* modifier in summer of the analysis year was the most consistent and critical soil variable for predicting the occurrence of fire in that year. The next most important modifier was soil water status in the previous year’s spring, explaining 30 % of the variance comparable with *fASW* in the following summer. Similar in predictive power was *fASW* in the current fall. Lastly, the previous winter and the previous fall conditions contributed some explanatory power to the model.

**Fig. 3:**
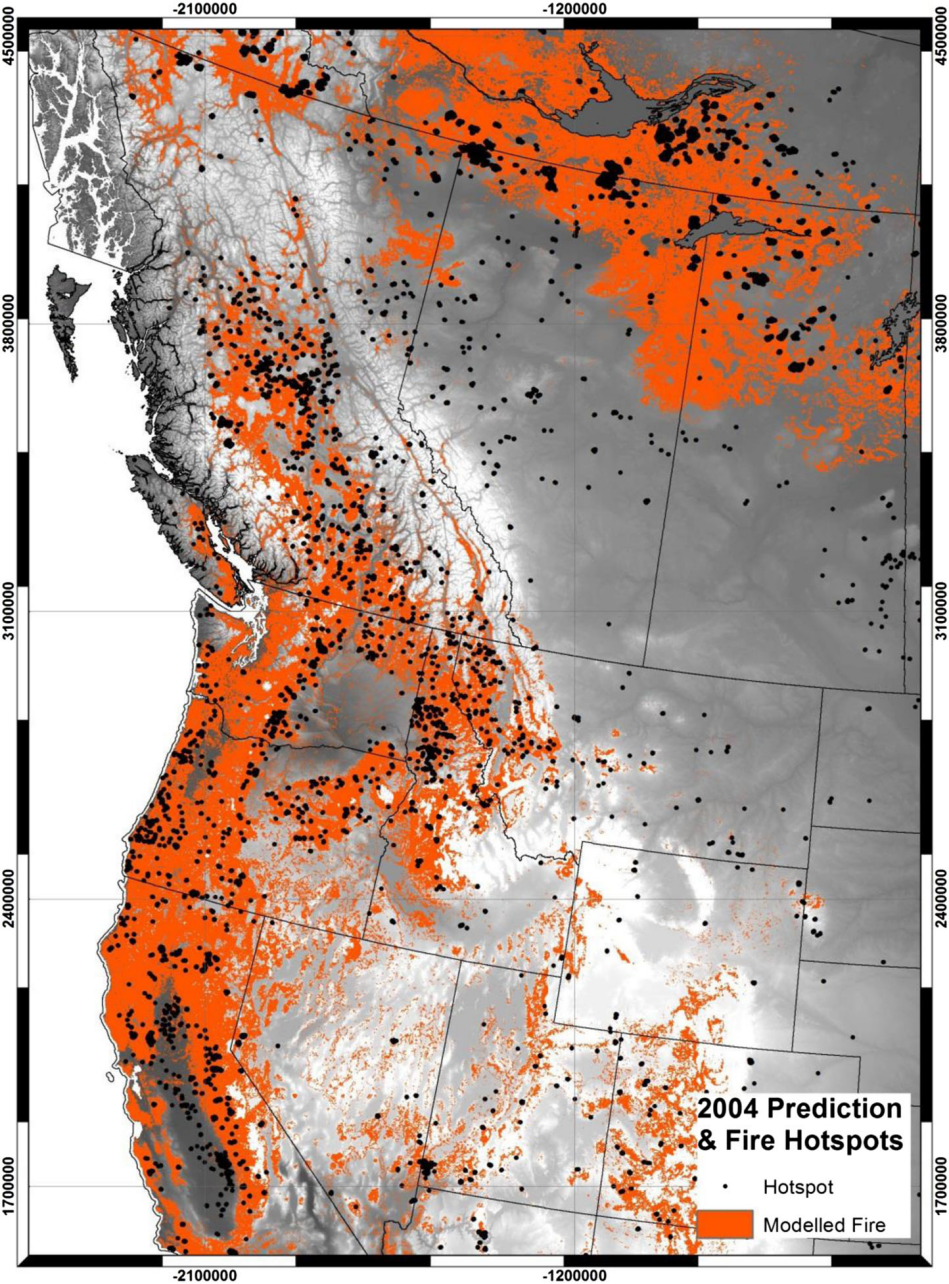
Model predictions of wildfires for 2004 (orange) in forested portions of western North America and the locations of MODIS active fire hotspots (black dots) for the same period

Figure 3 provides a graphic display of the area predicted by the model (in orange) to be susceptible to fire along with actual locations in 2004 (black dots). The active fires in the forested areas of the study area were closely correlated spatially to high, dry plateaus in British Columbia, Idaho and Montana as well as to more coastal areas in northern California and throughout most of central California. In contrast, the coastal areas of British Columbia, and northern Washington and Oregon had very few active fires recorded in 2004. In addition, fires occurred frequently in the northern locations of the study area, in the southern Boreal Forest Region, in Alberta and Saskatchewan. In general, the predicted areas correspond fairly well to the active fire points. Areas where fires were observed in 2004, but the model predicted none, include central and eastern Montana, some places in Nevada and in the eastern foothills of the Rocky Mountains in Alberta.

**Fig. 4:**
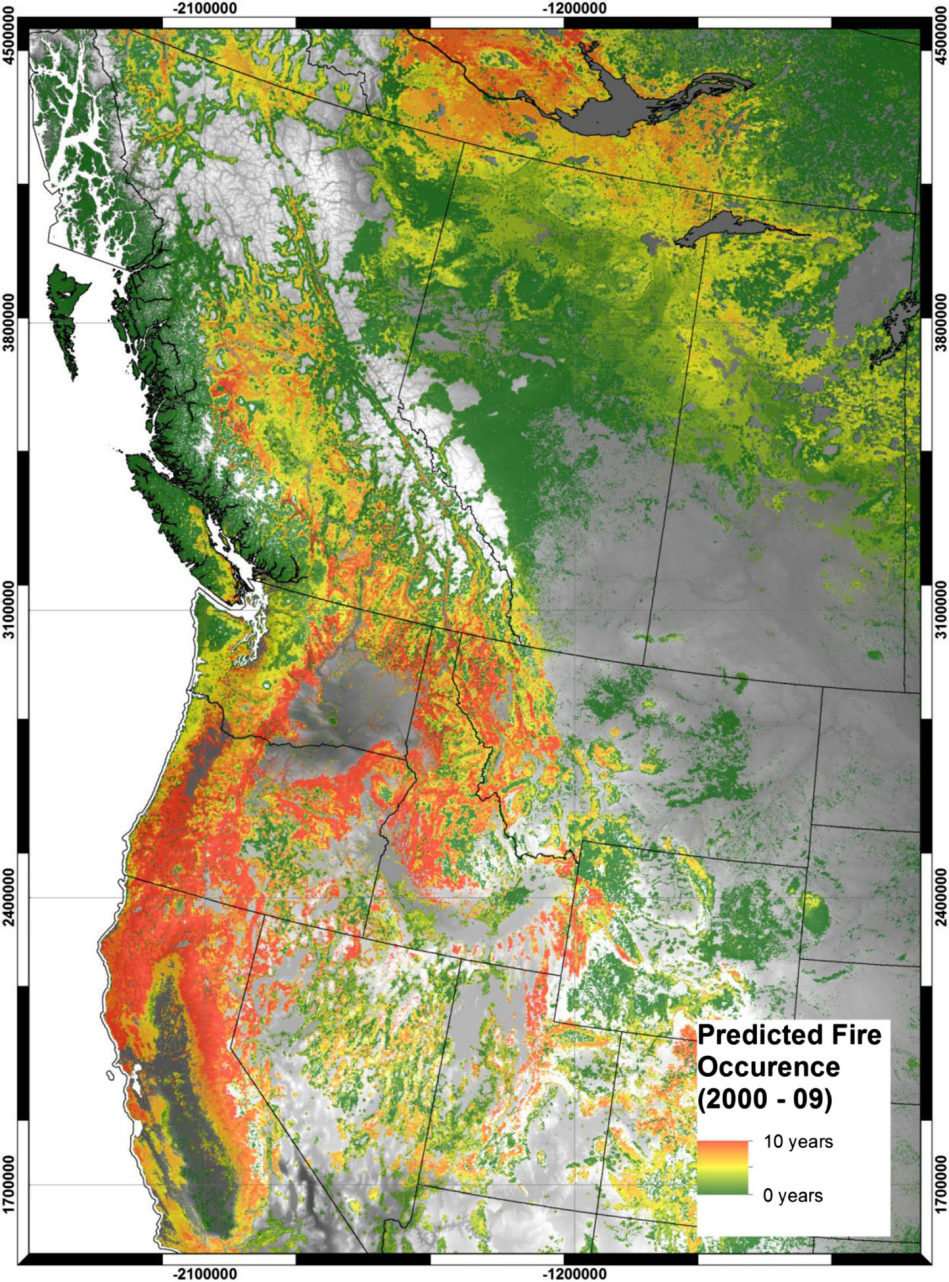
Accumulated predicted fire occurrence for the decade 2000–2009 in forested 1 km pixels based on the decision tree models

Figure 4 provides a decadal summation of the model predictions, showing the likelihood of fire occurrence over the decade from 2000 to 2009. The decadal map confirms the prevalence of fire in the forests of northern California, central Washington and Oregon, as well as the high plateau regions of British Columbia and the southern Boreal Region. The patterns differ somewhat from those indicated for a single year (Fig. 3) because outside of California, the location of the majority of predicted fires shifts considerably from year to year (maps not shown but are available online as 10 separate layers: http://databasin.org/datasets/78805401ae8e467b942f05e985742a14

**Fig. 5:**
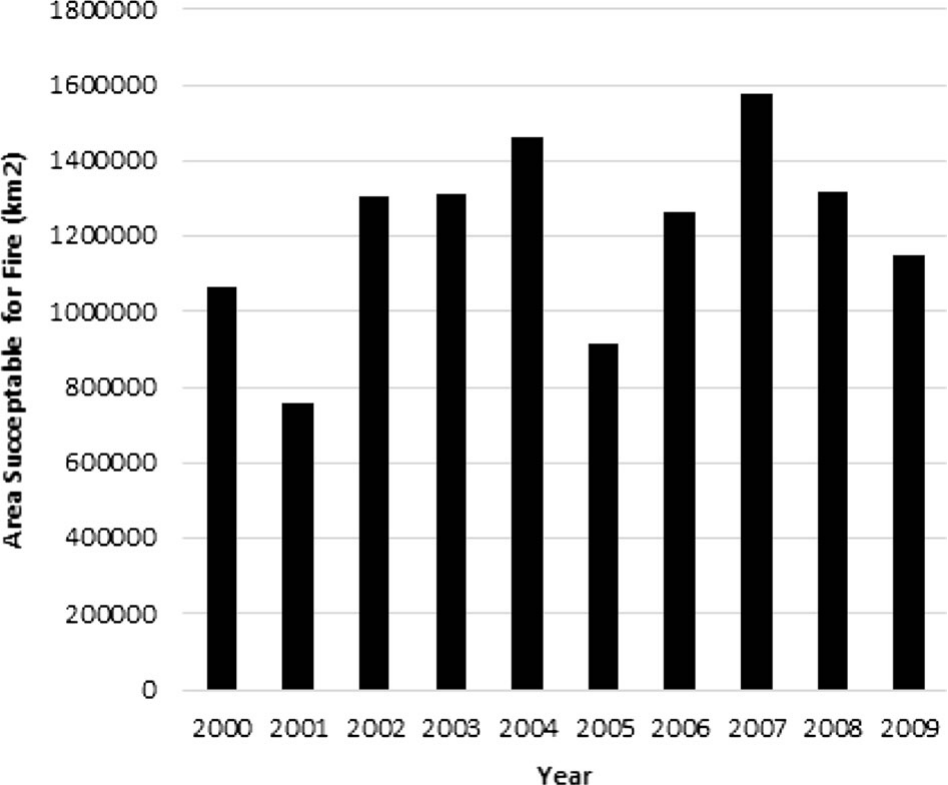
Area of fires predicted between 2000 and 2009 based on the decision tree model

Lastly, the area predicted as most susceptible to fire across the study area from 2000 to 2009 is shown in Fig. 5 and highlights fire-prone years (2002, 2004 and 2007); 2007 was modeled at almost 30 % above average and corresponded with one of the worst fire seasons on record in California. Conversely, some years were less prone to burn (2001, 2005 and 2009); 2001 was predicted to have 50 % less area burnt than the average for the decade. The model predicted 2000, 2006 and 2009 as representing average fire conditions for the period.

## Discussion

### Physiological insights

Even at a spatial resolution of 1 km, considerable variation occurs in topography, soils, and vegetation throughout the study area. Analyzing the occurrence of fire at 1 km subsumes much of this variation, increasing the probability that changes in weather conditions will account for much of the variation in predicting wildfires. It has been long recognized that LAI_max_ normally reflects a balance with the availability of soil water during seasons with potential deficits (Grier and Running 1977). A disequilibrium is established when the hydrologic conditions are significantly altered. Even in very open pinyon pine and juniper woodland, an exceptional drought is required to weaken the pine to the extent that they are susceptible to bark beetle attack (Breshears et al. 2009). Process-based forest growth models, like 3-PG, establish upper limits of transpiration as a function of whole canopy stomatal conductance and evaporative demand. Once LAI_max_ drops below ~5.0, the maximum rates of average daily transpiration fall from ~3.0 to <0.5 mm day^−1^ (Granier et al. 2000). It is important to recognize that dense forests exposed to high evaporative demand extract water much faster from the soil than more open stands; these differences compensates at times for variation in the AWS_max._ But good estimates of ASW_max_ are important. If all sites were assumed to have 200 mm at AWS_max_, those with LAI_max_ of 2.0 would require >3 months to draw down the supply to the extent that drought had any direct effect on stomata whereas those with an LAI_max_ of 6.0 would reach the same point in about a month (Running and Coughlan 1988). By focusing on seasonal anomalies in *f*(*ASW*) we were able to identify those conditions conducive to abnormally high production of ground fuel as well as those with highly flammable overstory vegetation.

### Improvements in modeling and data acquisition

Using every year of hot spot data from 2000 to 2009 could result in issues with temporal autocorrelation if the possibility was high that large areas could burn more than once in the decade. By selecting 2001, 2004, and 2007 we tried to minimize this issue by allowing at least 3 years for fuels to accumulate at a site.

It would be possible to extend the analysis back to the early 70s using Landsat imagery and to improve assessments of changes in forest structure henceforth by measuring the vertical distribution of LAI using airborne light detection and ranging (lidar) sensors (Lefsky et al. 2001; Bolton et al. 2015). More accurate estimates of LAI_max_ would permit derivation of more precise estimates of biomass in dense forest than is now possible. To cover larger areas, radar and hyperspectral data can be combined to obtain estimates of standing biomass, which would provide a basis for assessing changes in the amount and vertical distribution of potential fuels (Treuhaft et al. 2003).

At a spatial resolution of 1 km, model predictions of large wildfires begin to have policy implications (Kennedy and Johnson 2014; North et al. 2015). The possibility of prescribing fuel treatments in places where decision tree models or other types predict high probability of fire in the future would be a valuable application, one that would justify incorporating more detailed information on ASW_max_ and LAI_max_ in fire-prediction models.

### Implications for fire ecology and management

The approach introduced in this paper can identify areas where large fires may occur that could coalesce into mega fires (San-Miguel-Ayanz et al. 2013), and does so without recognizing regional or political boundaries. Almeida and Sands (2015) provide a new version of the 3-PG model with daily time-steps and other refinements that warrant testing during the active fire season. As presented, the approach reflects known fire histories quit well (69 % accuracy at 1 km resolution) and our understanding of how recurrent fires create a mosaic that define the distribution of forest types and their fuel characteristics. Understanding this pattern can inform forest restoration efforts so that they will be consistent with projected wildland fires.

Wildland fires can be expected to establish new landscape patterns over time, while correcting the “fire deficit” created following a century of fire exclusion (Marlon et al. 2012; North et al. 2015). The patterns are not expected to attain stability, however, because projected temperature increases, derived from 11 climate models, are expected to result in an increase in total cloud-to-ground lightning flashes of 12 % ± 5 % per degree Celsius of global warming, equivalent to a 50 % increase over the rest of this century for the contiguous United States (Romps et al. 2014). In some regions, such as southern California, most fires are human caused, either directly, or indirectly (Syphard and Keeley 2015). Regardless of the cause, fire is a catalyst for change in species distribution, migration, and extinction and ultimately may determine whether American forests remain a carbon sink in this century (Flannigan et al. 2000).
